# Expansion of healthcare-associated hypervirulent KPC-2-producing *Klebsiella pneumoniae* ST11/KL64 beyond hospital settings

**DOI:** 10.1016/j.onehlt.2023.100594

**Published:** 2023-06-26

**Authors:** Fernanda Esposito, Brenda Cardoso, Fábio P. Sellera, Elder Sano, Danny Fuentes-Castillo, Herrison Fontana, Bruna Fuga, Quézia Moura, Maria I.Z. Sato, Carlos J. Brandão, Nilton Lincopan

**Affiliations:** a Department of Clinical Analysis, School of Pharmacy, University of São Paulo, São Paulo, Brazil; b One Health Brazilian Resistance Project (OneBR), Brazil; c Department of Microbiology, Institute of Biomedical Sciences, University of São Paulo, São Paulo, Brazil; d Department of Internal Medicine, School of Veterinary Medicine and Animal Science, University of São Paulo, São Paulo, Brazil; e School of Veterinary Medicine, Metropolitan University of Santos, Santos, Brazil; f Departamento de Patología y Medicina Preventiva, Facultad de Ciencias Veterinarias, Universidad de Concepción, Chillán, Chile; g Federal Institute of Education Science and Technology of Espírito Santo, Vila Velha, Brazil; h Environmental Company of São Paulo State (CETESB), São Paulo, Brazil

**Keywords:** Genomic surveillance, WHO critical priority pathogens, Enterobacterales, Virulome, Resistome, Carbapenems, One Health, Environmental dissemination

## Abstract

The spread of carbapenemase-producing *Klebsiella pneumoniae* beyond hospital settings is a global critical issue within a public health and One Health perspective. Another worrisome concern is the convergence of virulence and resistance in healthcare-associated lineages of *K. pneumoniae* leading to unfavorable clinical outcomes. During a surveillance study of WHO critical priority pathogens circulating in an impacted urban river in São Paulo, Brazil, we isolate two hypermucoviscous and multidrug-resistant *K. pneumoniae* strains (PINH-4250 and PINH-4900) from two different locations near to medical centers. Genomic investigation revealed that both strains belonged to the global high-risk sequence type (ST) ST11, carrying the *bla*_KPC-2_ carbapenemase gene, besides other medically important antimicrobial resistance determinants. A broad virulome was predicted and associated with hypervirulent behavior in the *Galleria mellonella* infection model. Comparative phylogenomic analysis of PINH-4250 and PINH-4900 along to an international collection of publicly available genomes of *K. pneumoniae* ST11 revealed that both environmental strains were closely related to hospital-associated *K. pneumoniae* strains recovered from clinical samples between 2006 and 2018, in São Paulo city. Our findings support that healthcare-associated KPC-2-positive *K. pneumoniae* of ST11 clone has successfully expanded beyond hospital settings. In summary, aquatic environments can become potential sources of international clones of *K. pneumoniae* displaying carbapenem resistance and hypervirulent behaviors, which is a critical issue within a One Health perspective.

## Highlights

• WHO critical-priority *K. pneumoniae* strains were recovered from urban rivers.
• Genomic data revealed presence of global hospital-associated clones KPC-2/ST11.
• Environmental hypermucoviscous KPC-2/ST11 carried broad resistomes/virulomes.
• Phylogenomics revealed clonal relatedness with human and environmental lineages.
• Expansion of KPC-2/ST11 clones beyond hospital walls is a critical One Health issue.

## Graphical abstract

**Figure:**
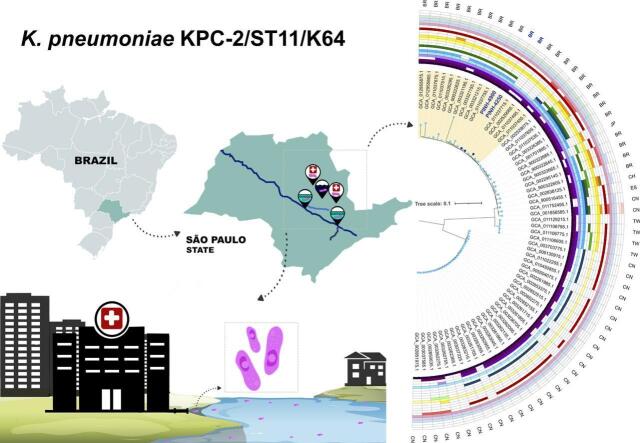

## 1 Introduction

The environmental dissemination of carbapenem-resistant *Klebsiella pneumoniae* (CRKp) is a critical public health and One Health issue [1]. In fact, due its clinical impacts and therapeutic challenges, CRKp has been recently classified as a critical priority pathogen by the WHO [2,3], with carbapenem resistance being mostly associated with the production of carbapenemases such as KPC-2, NDM-1, and OXA-232 [4]. In the last years, hypervirulent *K. pneumoniae* (hvKp) strains displaying hypermucoviscosity (hmKp) have emerged in Asia, Europe and South America, being associated to capsular serotypes (K) and sequence type (ST) combinations K1/ST23, K2/ST86, K2/ST65, K16/ST685, K20/ST268, K20/ST420, K54/ST29, K57/ST41 and K57/ST218 [[4], [5], [6], [7]], all of them causing a variety of infections with significant rates of morbidity and mortality [8]. It is noteworthy that, while most hvKp strains exhibit an antibiotic-sensitive profile [9], CRKp strains display a relatively low virulent behavior. However, convergence of hypervirulence and carbapenem-resistance in *K. pneumoniae* has emerged in hospital settings [4,7], which requires close monitoring, in order to avoid its environmental spread. Utilizing a microbiological and whole genome sequencing methodology, we have undertaken a surveillance investigation to examine the prevalence of pathogenic bacteria harboring clinically relevant resistance genes in anthropogenically-impacted urban rivers in Brazil. In this regard, we report that healthcare-associated hypervirulent KPC-2-producing *K*. *pneumoniae* ST11/KL64 has successfully expanded beyond hospital settings, which constitute a critical issue within a One Health perspective.

## 2 Materials and methods

### 2.1 Water samples collection, bacterial identification, and antimicrobial susceptibility testing

During a Brazilian surveillance study (OneBR project), we investigated the spread of WHO critical priority pathogens circulating in urban rivers in São Paulo, the most populated city in South America. In this regard, from 2016 to 2018, 500 mL of surface water samples were collected from three different locations at the Pinheiros River (−23.702500: −46.673889;-23.664722: −46.709722; and − 23.531111, −46.748333), and kept refrigerated until being processed (within 6 h after collection). In brief, 100 mL of each sample was filtered by 0.45 μm Millipore membrane. Subsequently, the membranes were placed onto MacConkey agar plates supplemented with meropenem (2 μg/mL) or ceftriaxone (2 μg/mL) [10].

Meropenem- and/or ceftriaxone-resistant strains were identified by matrix-assisted laser desorption ionization–time of flight mass spectrometry (MALDI-TOF MS) [11]. Antimicrobial susceptibility was performed using veterinary and human antibiotics [12,13], including amoxicillin-clavulanic acid, ceftiofur, ceftriaxone, cefotaxime, ceftazidime, cefepime, aztreonam, cefoxitin, ertapenem, imipenem, meropenem, nalidixic acid, amikacin, gentamicin, tobramycin, ciprofloxacin, levofloxacin, enrofloxacin, tetracycline, fosfomycin, chloramphenicol, colistin and trimethoprim/sulfamethoxazole. Antibiotics were tested by disk diffusion (Kirby-Bauer) method, except colistin, which was assessed using the broth microdilution method to determine the minimum inhibitory concentration (MIC). The interpretations were carried out according to the Clinical and Laboratory Standards Institute recommendations [12,13]. Meropenem- and/or ceftriaxone-resistant strains were subjected to whole genome sequence (WGS) analysis.

### 2.2 Whole genome sequence analysis

Genomic DNA of bacterial strainswere extracted using PureLink Quick Gel Extraction Kit (Life Technologies, Carlsbad, CA). Whole genome sequencing of PINH-4250 and PINH-4900 were performed using a MiSeq (2 × 150-bp paired-end) and NextSeq550 (2 × 75-bp paired-end) platforms (Illumina), respectively. Raw sequencing data with a PHRED quality score below 20 were removed using TrimGalore v0.6.5 (https://github.com/FelixKrueger/TrimGalore). *De novo* genome assembly was performed using default parameters of Unicycler v0.4.8. (https://github.com/rrwick/Unicycler), and genomes annotation were carried out using the NCBI Prokaryotic Genome Annotation Pipeline (PGAP) v. 3.2 (http://www.ncbi.nlm.nih.gov/genome/annotation_prok/).

Multi-locus Sequence Typing (MLST) prediction was performed using MLST v.2.0 (https://cge.cbs.dtu.dk/services/MLST/). Virulomes were predicted by using theVFDB–Virulence Factor Database (https://github.com/haruosuz/vfdb) and BIGSdb database for *K. pneumoniae* (http://bigsdb.pasteur.fr/klebsiella/klebsiella.html). A > 95% identity threshold was applied as a filtering criterion for identification of all predicted genes. Kleborate was used to predict integrative conjugative element (ICE) associated with virulence loci [ICEKp – colibactin (*clb*), yersiniabactin (*ybt*)], O antigen (LPS) and K (capsule) serotypes [14].

The plasmidome and resistome of bacterial strains were predicted by PlasmidFinder 2.0 (https://cge.food.dtu.dk/services/PlasmidFinder/) and ResFinder 4.1 (https://cge.food.dtu.dk/services/ResFinder/), respectively, whereas the presence of heavy metal (HM) genes encoding, and biocides tolerance were manually identified using the ABRicate v0.9.8 (https://github.com/tseemann/abricate) through BacMet (http://bacmet.biomedicine.gu.se/blast/blast_link.cgi) and our *in-house* database.

### 2.3 Phylogenomic analysis of healthcare-associated and environmental *K. pneumoniae* ST11

For comparative genome analysis, we performed a search for *K. pneumoniae* ST11 genomes on BacWGSTdb (http://bacdb.cn/BacWGSTdb). All genomes with data for country, isolation source and collection year were downloaded from NCBI Genbank (*n* = 962). Since K64 isolates form a distinct clade among *K. pneumoniae* ST11 strains [15], Kaptive v2.0.0 (https://github.com/katholt/Kaptive) was used to assess K-locus and select only K64 isolates (*n* = 333), including close isolates with low coverage and/or genes missing. FastANI v1.32 (https://github.com/ParBLiSS/FastANI) was used to assess average nucleotide identity (ANI) among the 333 K64 genomes and each *K. pneumoniae* strain identified in this study. The 50 genomes with highest ANI with each strain were selected for phylogenetic analysis, totalizing 60 genomes. Then, we generated a SNP-based maximum-likelihood phylogenetic tree with *K. pneumoniae* strains isolated from Pinheiros River, and the 60 selected genomes using default settings of CSI Phylogeny v1.4 (https://cge.cbs.dtu.dk/services/CSIPhylogeny), which also generated a SNP distance matrix. Chromosome sequence of *K. pneumoniae* ST11 strain KPC160121 (RefSeq accession number: NZ_CP040028.1) was used as reference. ABRicate v1.0.1 (https://github.com/tseemann/abricate) was used with ResFinder (https://bitbucket.org/genomicepidemiology/resfinder_db) and PlasmidFinder (https://bitbucket.org/genomicepidemiology/plasmidfinder_db) to identify plasmid replicons and antibiotic resistance genes in all genomes in the tree. We also used ABRicate with an *in-house* built database for identifying heavy metal and biocide resistance genes, and a database built from Institut Pasteur BIGSdb *Klebsiella* (https://bigsdb.pasteur.fr/klebsiella) virulence scheme on allele profiles database for identifying virulence genes. Coverage and identity thresholds were set to 100 and 98% respectively, on all ABRicate analyses. Kleborate v2.2.0 (https://github.com/katholt/Kleborate) was used to identify virulence genes and mutations on quinolone resistance determining regions (QRDR). The tree was midpoint-rooted using iTOL v6 (https://itol.embl.de), which was also used to annotate the three with data from BacWGSTdb, ABRicate, and Kleborate databases.

### 2.4 Hypermucoviscosity test and genomic background of K-locus

Hypermucoviscosity phenotype of *K. pneumoniae* strains were analyzed by the string test, which is the ability of bacterial colonies grown on agar plates to form viscous strings of >5 mm when stretched is strongly associated with hypervirulence (hypermucoviscous) in *K. pneumoniae* pathogens [6,[16], [17], [18]]. The inference of capsule structure was performed using BLASTn, followed by manual curation using Geneious Prime version. 2022.1.1 (Biomatters, New Zealand), against the K64 *K. pneumoniae* NCTC 8172 reference genome (GenBank accession number: AB924600.1) [19].

### 2.5 *In vivo* virulence assays of environmental *K. pneumoniae* ST11

The virulence potential of environmental *K. pneumoniae* strains were assessed by using the greater wax moth (*Galleria mellonella*) infection model [16,20]. In this respect, *G. mellonella* is a low-cost invertebrate infection model that presents an innate immune system highly similar with that of mammals, including cellular and humoral response [21]. In brief, groups of ten *G. mellonella* larvae of nearly 250 to 350 mg were infected with a10 μL aliquot containing10^6^ CFU/mL of each strain, and survival was monitored for 96 h. Two biological replicates and two technical replicates were conducted to ensure experimental reproducibility. The hypervirulent *K. pneumoniae* (hvKP) K1/ST23 strain A58300 and the non-virulent *K. pneumoniae* ATCC 13883 were used as comparative controls [22]. Statistical analysis was conducted using the log-rank test, while the Kaplan-Meier method was employed for plotting survival curves. Through Graph Pad Software (San Diego, CA, USA) [16,20].

## 3 Results and discussion

In this study, two carbapenem-resistant *K. pneumoniae* strains (PINH-4250 and PINH-4900) were recovered from two different locations (−23.664722: −46.709722 and − 23.531111: −46.748333) at the Pinheiros River, which runs 25 km (16 miles) through the city of São Paulo. Pinheiros River has been subjected to persistent anthropogenic pollution stemming from the continuous discharge of untreated domestic sewage into its various tributaries. Furthermore, inadequate street sweeping practices and the contamination of soils by industrial runoff and discharges contribute to the contamination of this river. In accordance with Resolution 357/2005 of the Brazilian Environment National Council (CONAMA), which classifies water quality into five categories ranging from pristine to polluted, according to the qualities that are required for their preponderant use, the Pinheiros River falls under class 4 classification, indicating a high level of pollution [23].

PINH-4250 and PINH-4900 strains displayed a multidrug-resistant (MDR) profile to amoxicillin-clavulanic acid, cefotaxime, ceftriaxone, ceftiofur, ceftazidime, cefepime, cefoxitin, aztreonam, ertapenem, imipenem, meropenem, nalidixic acid, amikacin, gentamicin, ciprofloxacin, levofloxacin, enrofloxacin, chloramphenicol, and trimethoprim/sulfamethoxazole. In addition, environmental PINH-4900 strain was also resistant to tetracycline and tobramycin.

**Fig. 1:**
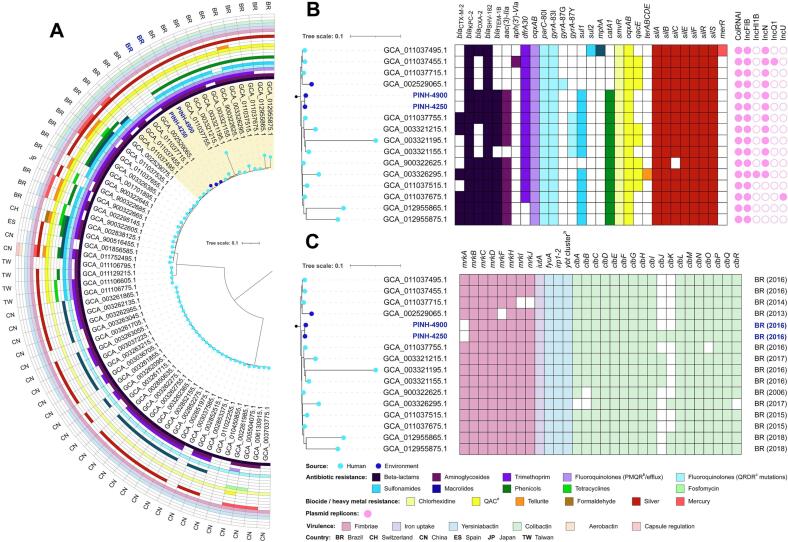
In A, SNP-based phylogenetic tree of 60 *Klebsiella pneumoniae* ST11 globally identified from human and environment, containing their source and country of isolation. In B and C, the highlighted clade subtree presents the 14 closely related genomes to PINH-4250 (ONE211) and PINH-4900 (ONE271) strains. In B, heatmap displaying the resistome and plasmidome of *K. pneumoniae* cluster. In C, heatmap displaying the virulome of the *K. pneumoniae* cluster. ^a^*ybt* cluster: *ybtSXQPAUTE*; ^b^PMQR: Plasmid Mediated Quinolone Resistance; ^c^QRDR: Quinolone Resistance Determining Region; ^d^QAC: Quaternary Ammonium Compound.

Both environmental strains were assigned to ST11, which has been recognized as a globally widespread lineage, being recovered from human and animal hosts, in several countries, including Brazil, Colombia, Egypt, Switzerland, Spain, Pakistan, Japan, Taiwan, and China. In this context, among the 60 *K. pneumoniae* ST11 genomes selected, phylogenomic analysis revealed a SNP range difference between 0 and 2313 (Supplementary Table 1); whereas ANI ranging varied between 99,3145 and 99,9762% (Fig. 1A; Supplementary Table 2).

It is noteworthy that, environmental PINH-4250 and PINH-4900 strains were closely related to 14 clinical strains [24], and another environmental strain isolated from a water sample from an urban lake [25], which were recovered between 2006 and 2018, in Brazil (Fig. 1A). These findings are worrisome, since they support an anthropogenic origin of the spread of high-risk clones of *K. pneumoniae* for aquatic environments, impacting the human-environmental connectivity.

PINH-4250 and PINH-4900 strains carried a broad resistome, which consisted of genes conferring resistance to β-lactams (*bla*_KPC-2_, *bla*_OXA-2_, *bla*_SHV-11_, and *bla*_TEM-1D_), aminoglycosides [*aac(3′)-Iia*], macrolide (*mphA*), sulphonamides (*sul1*), phenicols (*catA1*), fosfomycin (*fosA*), and trimethoprim (*dfrA30*) (Table 1; Fig. 1B). Chromosomal point mutations in *parC* (S80I) and *gyrA* (S83L) were detected. On the other hand, the *tetA* gene, associated with tetracycline resistance, was exclusively detected in the PINH-4900 strain. Moreover, genes associated with resistance to silver (*silABCEFRS*), chlorhexidine (*smvR*) and quaternary ammonium compounds (*oqxAB*) were also detected, in both PINH-4250 and PINH-4900 environmental strains. Furthermore, plasmidome analysis demonstrated the presence of IncN1, IncFIB, and ColRNAI plasmids (Table 1; Fig. 1B).

**Table 1:** Genomic features of carbapenem-resistant *Klebsiella pneumoniae* strains isolated from the Pinheiros River, Brazil.

Characteristics	PINH-4250	PINH-4900
Source	Urban river water	Urban river water
Year of isolation	2016	2016
Genome size (bp)	5,835,231	5,813,875
G + C content (%)	57,1	57
rRNA	4	3
tRNAs	81	41
ncRNAs	11	6
N° total of genes	5812	5829
No. of CDS^a^	5715	5779
ST/CG^b^	11/258	11/258
K-locus/O-locus	KL64/O2v1	KL64/O2v1
*wzi*	64	64
Virulome	*ybtSXQPAUTE*, *irp1–2*, *fyuA*, *clbABCDEFGHILMNOPQ*, *iutA*, *mrkBCDFHIJ*	*ybtSXQPAUTE, irp1–2*, *fyuA*, *clbABCDEFGHIJKLMNOPQ*, *iutA*, *mrkBCDFHIJ*
Resistome		
Antibiotics		
β-Lactams	*bla*_KPC-2_, *bla*_OXA-2_, *bla*_SHV-11_, *bla*_TEM-1D_	*bla*_KPC-2_, *bla*_OXA-2_, *bla*_SHV-11_, *bla*_TEM-1D_
Aminoglycosides	*aac(3′)-IIa*	*aac(3′)-IIa*
Quinolones	*gyrA*-83I, *parC*-80I	*gyrA*-83I, parC-80I
Macrolides	*mphA*	*mphA*
Sulfonamides	*sul1*	*sul1*
Tetracycline	–	*tetA*
Trimethoprim	*dfrA30*	*dfrA30*
Chloramphenicol	*catA1*	*catA1*
Fosfomycin	*fosA*	*fosA*
Heavy metal		
Silver	*silABCEFRS*	*silABCEFRS*
Biocides		
QACs^c^	*smvR, oqxA, oqxB*	*smvR, oqxA*, *oqxB*
Plasmids	IncN1, IncFIB, ColRNAI	IncN1, IncFIB, ColRNAI
OneBR ID	ONE211	ONE271
GenBank accession	JAEDYS000000000	JAECUX000000000

a CDSs, coding sequences.

b ST, sequence type; CG, clonal group.

c QACs, Quaternary ammonium compounds.

The virulome of environmental PINH-4250 and PINH-4900 strains consisted of *irp-1-2,* the operon *ybtAEPQSTU*X (yersiniabactin siderophore synthesis), *fyuA* (yersiniabactin receptor), *iutA* (iron uptake), *clb* genes(colibactin genotoxin synthesis), and the *mrkBCDFHIJ* cluster (type 3 fimbrial synthesis) (Table 1; Fig. 1C), which have been related to human infections associated with unfavorable outcomes, and with the pathogen survival in the respiratory tract [[26], [27], [28]]. Additionally, both *K. pneumoniae* strains comprised an integrative conjugative element ICEKp10, O2v1 locus, and K64/*wzy*-64 capsule type.

**Fig. 2:**
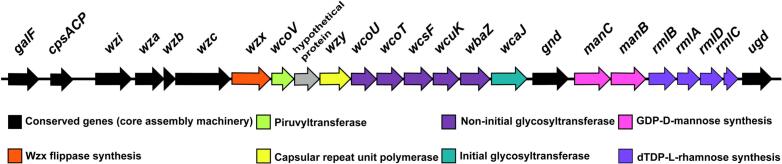
K64 capsule structure of environmental *K. pneumoniae* PINH-4250 and PINH-4900 strains. Conserved genetic organization involved with core assembly machinery are represented in black arrows. The *wzc*-*gnd* region was consist to genes associated with synthesis of flippase [*wzx*; orange arrow]; piruvyl tranferase [*wcoV*; light-green arrow]; polymerase [*wzy*; yellow arrow]; non-initial [*wcoUT*, *wcsF*, *wcuK* and *wbaZ*; purple arrows]; and initial [*wcaJ*; green arrow] glycosyl transferase. The *gnd*-*ugd* region comprised genes involved in GDP-D-mannose synthesis [*manB* and *manC*; pink arrows] and deoxythymidine diphosphate dTDP-L-rhamnose synthesis [*rmlA*, *rmlB*, *rmlC* and *rmlD*; violet arrows]. (For interpretation of the references to colour in this figure legend, the reader is referred to the web version of this article.)

Capsule structure analysis revealed a conserved genetic organization involved with core assembly machinery (*galF* to *wzc* genes), at the 5′ end of the *cps* locus; whereas the *wzc*-*gnd* region was consist to genes associated with flippase (*wzx*), piruvyl tranferase (*wcoV*), polymerase (*wzy*), non-initial (*wcoUT*, *wcsF*, *wcuK* and *wbaZ*) and initial (*wcaJ*) glycosyltransferase, as previously reported [19]. Furthermore, the *gnd-ugd* region encompassed *manB* and *manC* genes, which are associated with the biosynthesis of GDP-D-mannose; as well as *rmlA, rmlB*, *rmlC* and *rmlD* genes, that are responsible for deoxythymidine diphosphate dTDP-L-rhamnose synthesis (Fig. 2). In this regard, D-mannose and L-rhamnose saccharides plays an important role in the synthesis of adhesins involved in the process of pathogen-pathogen and pathogen-host interaction and immune evasion of different pathogenic bacteria and fungi [[29], [30], [31], [32], [33]].

HvKp strains with a positive string test frequently display a high invasiveness behavior and have been associated with severe infections such as bacteremia, metastatic endophthalmitis, osteomyelitis, meningitis, septic arthritis, and muscle, epidural and liver abscesses [8,34,35]. Although neither of the known genes encoding hypermucoviscosity (*rmpA* or *rmpA2* or *magA*) were detected in the genome of PINH-4250 and PINH-4900 (Fig. 2B-C), both strains displayed a hypermucoviscous (HMV) phenotype. In fact, previous studies also reported *rmpA*−/HMV + *K. pneumoniae* clinical strains belonging to different serotypes and ST such as K31/ST104, K30/ST234, K3/ST321, K35/ST460 and K21/ST1007 [17].

**Fig. 3:**
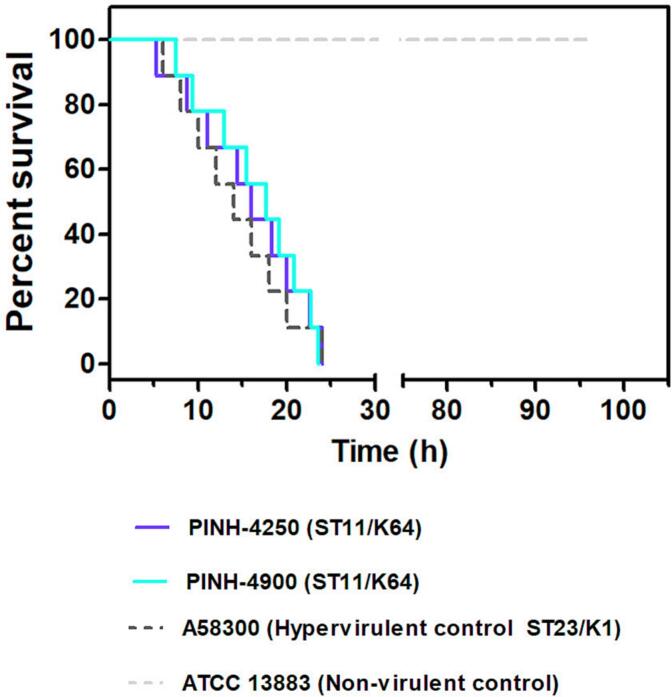
Virulent behavior of environmental K64/ST11/KPC-2-producing PINH-4250 and PINH-4900 strains. Survival curves of *Galleria mellonella* infected with 10^6^ CFU/larva of *K. pneumoniae* PINH-4250 strain (violet line), *K. pneumoniae* PINH-4900 strain (light-green line), *K. pneumoniae* A58300 strain (dark-grey dashed line) and *K. pneumoniae* ATCC 13883 strains (light-grey dashed line). Environmental PINH-4250 and PINH-4250 isolates, and the clinical K1/ST23 A58300 strain killed 100% of larvae at 24 h post-infection. On the other hand, the control group inoculated with the ATCC 13883 strain showed 100% survival. Each replicate was conducted using groups containing 10 *G. mellonella* larvae per strain. Two biological replicates and two experimental replicates were performed. (For interpretation of the references to colour in this figure legend, the reader is referred to the web version of this article.)

Although *G. mellonella* is not a natural host for *K. pneumoniae*, it has been effectively employed as an infection model to evaluate the pathogenicity of *K. pneumoniae* strains, owing to its resemblance to vertebrates in terms of the innate immune system [16,20,21]. In this regard, the hypervirulent behavior of K64/ST11 PINH-4250 and PINH-4900 strains was proven in the greater wax moth infection model. Indeed, both strains were capable to kill 100% larvae at 24 h post-infection, which was identically to the hypervirulent K1/ST23 control strain A58300 (Fig. 3) [22]. This hypervirulent behavior of K64/ST11 clones has been previously reported in human infections related to outbreaks in China [6,36].

In summary, we report the successful expansion of the healthcare-associated and hypervirulent KPC-2-producing *K. pneumoniae* ST11/KL64 to an anthropogenically impacted river in Brazil. Historically, this river has suffered for a long time from several sources of pollution caused by anthropogenic activities, such as solid waste discharges, industrial runoff, and daily released non-treated domestic sewage on the various tributaries [23]. Strikingly, previous studies have reported the occurrence of CTX-M-15 or KPC-2-producing *K. pneumoniae* isolates belonging to global clones ST15, ST437, ST11, ST340 or ST321, in urban lakes and rivers, in Brazil Portugal and Switzerland [25,[37], [38], [39]]. Currently, aquatic environments are recognized as important reservoirs and hot spots for clinically significant MDR bacteria and antibiotic resistance genes [40]. In fact, the WHO and the International Water Association (IWA) have classified the aquatic environments as vehicles for the sharing and acquisition of bacteria carrying medically important resistance genes, such as the *bla*_KPC-2_ gene [41]. Specifically, for the dissemination of carbapenemase-positive bacteria, several studies have demonstrated an epidemiological link with anthropogenic activities, including discharge of domestic and/or hospital wastewater effluents that did not receive appropriate treatment [38,[41], [42], [43], [44], [45]]. Therefore, the assessment of water quality must include the examination of the multidrug resistance of clinically relevant bacterial species, providing an important link regarding the spread of MDR pathogens in a One Health context. In this regard, in this study, we demonstrate a One Health link based on a genomic approach, which reveal phylogenomic relatedness (55–93 SNP differences) between environmental and hospital-associated KPC-2-poducing *K. pneumoniae* strains of ST11, recovered between 2013 and 2017, and stability and adaptation of this lineage to impacted urban rivers. Interestingly, lowest SNP differences are observed among environmental and nosocomial strains isolated in the same year, confirming hospital origin of environmental ST11.

Our findings highlight the role of aquatic environments in spreading hypervirulent and carbapenem-resistant *K. pneumoniae*, since water systems could favor and escalating the emergence of other pathogens with such coexistence profiles, associated with untreatable invasive human and non-human infections, which constitutes an unprecedented major public health challenge under a One Health perspective. Therefore, efforts to expand and strengthen genomic investigation of WHO critical priority pathogens in aquatic environments subjected to anthropogenic pressures are necessary for effective surveillance programs.

The following are the supplementary data related to this article.

Supplementary Table 1 SNP counts for *Klebsiella pneumoniae* ST11 isolates. Supplementary Table 1

Supplementary Table 2 ANI counts for *Klebsiella pneumoniae* ST11 isolates. Supplementary Table 2

## Author cContributions

FE, BC, ES, and HF conducted the data analysis. FE, BC, QM, BF, and DF-C carried out the experimental procedures. MIZS and CJB were responsible for the collection, storage, and transportation of water samples. NL supervised the experiments, designed, and coordinated the project. FE, BC, FS, and NL contributed to the writing, reviewing, and editing of the manuscript. All authors made significant contributions to the article, approved the final version, and gave their consent for submission.

## Funding

This study received financial support from the 10.13039/100000865Bill and Melinda Gates Foundation (Grand Challenges Explorations Brazil OPP1193112). In accordance with the grant conditions of the Foundation, the author accepted manuscript version resulting from this submission is subject to a CC BY or equivalent license. Furthermore, the study was supported by the 10.13039/501100001807FAPESP (2020/08224-9 and 2019/15578-4) and 10.13039/501100003593CNPq (88882.333054/2019-01). FE was a FAPESP research fellow (2019/15578-4). BC and HF were CAPES research fellows (88882.333054/2019-01 and 88887.506496/2020-00), while BF was a PNPD/CAPES research fellow (88887.358057/2019-00). NL is a CNPq research fellow (314336/2021-4).

## Declaration of Competing Interest

All authors declare no conflicts of interest.

## Data Availability

The datasets presented in this study can be found in online repositories. Both PINH-4250 and PINH-4900 genome shotgun data have been deposited at GenBank database under the accession JAEDYS000000000 and JAECUX000000000, respectively. Additionally, genomic information of PINH-4250 and PINH-4900 K. pneumoniae strains are available on the OneBR – KpBr platform (http://onehealthbr.com/) under the number ID ONE211 and ONE271, respectively.

## References

[1] Antimicrobial Resistance Collaborators (2022). Global burden of bacterial antimicrobial resistance in 2019: a systematic analysis. Lancet.

[2] Wei T., Zou C., Qin J., Tao J., Yan L., Wang J. (2022). Emergence of hypervirulent ST11-K64 *Klebsiella pneumoniae* poses a serious clinical threat in older patients. Front. Public Health.

[3] Tacconelli E., Carrara E., Savoldi A., Harbarth S., Mendelson M., Monnet D.L. (2018). Discovery, research, and development of new antibiotics: the WHO priority list of antibiotic-resistant bacteria and tuberculosis. Lancet Infect. Dis..

[4] Mataseje L.F., Boyd D.A., Mulvey M.R., Longtin Y. (2019). Two Hypervirulent *Klebsiella pneumoniae* isolates producing a *bla*_KPC-2_ carbapenemase from a Canadian patient. Antimicrob. Agents Chemother..

[5] Du P., Zhang Y., Chen C. (2018). Emergence of carbapenem-resistant hypervirulent *Klebsiella pneumoniae*. Lancet Infect. Dis..

[6] Gu D., Dong N., Zheng Z., Lin D., Huang M., Wang L. (2018). A fatal outbreak of ST11 carbapenem-resistant hypervirulent *Klebsiella pneumoniae* in a Chinese hospital: a molecular epidemiological study. Lancet Infect. Dis..

[7] Yu F., Lv J., Niu S., Du H., Tang Y.W., Pitout J. (2018). Multiplex PCR analysis for rapid detection of *Klebsiella pneumoniae* carbapenem-resistant (sequence type 258 [ST258] and ST11) and hypervirulent (ST23, ST65, ST86, and ST375) strains. J. Clin. Microbiol..

[8] Choby J.E., Howard-Anderson J., Weiss D.S. (2020). Hypervirulent *Klebsiella pneumoniae* - clinical and molecular perspectives. J. Intern. Med..

[9] Xie M., Yang X., Xu Q., Ye L., Chen K., Zheng Z. (2021). Clinical evolution of ST11 carbapenem resistant and hypervirulent *Klebsiella pneumoniae*. Commun. Biol..

[10] Turano H., Gomes F., Medeiros M., Oliveira S., Fontes L.C., Sato M.I. (2016). Presence of high-risk clones of OXA-23-producing *Acinetobacter baumannii* (ST79) and SPM-1-producing *Pseudomonas aeruginosa* (ST277) in environmental water samples in Brazil. Diagn. Microbiol. Infect. Dis..

[11] Clark A.E., Kaleta E.J., Arora A., Wolk D.M. (2013). Matrix-assisted laser desorption ionization–time of flight mass spectrometry: a fundamental shift in the routine practice of clinical microbiology. Clin. Microbiol. Rev..

[12] Clinical and Laboratory Standards Institute (CLSI) (2021). Performance Standards for Antimicrobial Disk and Dilution Susceptibility Tests for Bacteria Isolated from Animals.

[13] Clinical and Laboratory Standards Institute (CLSI) (2022).

[14] Wyres K.L., Nguyen T., Lam M., Judd L.M., van VinhChau N., Dance D. (2020). Genomic surveillance for hypervirulence and multi-drug resistance in invasive *Klebsiella pneumoniae* from south and Southeast Asia. Genome Med..

[15] Zhao J., Liu C., Liu Y., Zhang Y., Xiong Z., Fan Y. (2020). Genomic characteristics of clinically important ST11 *Klebsiella pneumoniae* strains worldwide. J. Glob. Antimicrob. Resist..

[16] Moura Q., Esposito F., Fernandes M.R., Espinoza-Muñoz M., Souza T.A., Santos S.R. (2017). Genome sequence analysis of a hypermucoviscous/hypervirulent and MDR CTX-M-15/K19/ST29 *Klebsiella pneumoniae* isolated from human infection. Pathog. Dis..

[17] Cubero M., Grau I., Tubau F., Pallarés R., Dominguez M.A., Liñares J. (2016). Hypervirulent *Klebsiella pneumoniae* clones causing bacteremia in adults in a teaching hospital in Barcelona, Spain (2007-2013). Clin. Microbiol. Infect..

[18] Shon A.S., Bajwa R.P., Russo T.A. (2013). Hypervirulent (hypermucoviscous) *Klebsiella pneumoniae*: a new and dangerous breed. Virulence.

[19] Pan Y.J., Lin T.L., Chen C.T., Chen Y.Y., Hsieh P.F., Hsu C.R. (2015). Genetic analysis of capsular polysaccharide synthesis gene clusters in 79 capsular types of *Klebsiella* spp. Sci. Rep..

[20] Cardoso B., Esposito F., Fontana H., Fuga B., Moura Q., Sano E. (2022). Genomic analysis of a Kpi (pilus system)-positive and CTX-M-15-producing *Klebsiella pneumoniae* belonging to the high-risk clone ST15 isolated from an impacted river in Brazil. Genomics..

[21] Ménard G., Rouillon A., Cattoir V., Donnio P.Y. (2021). *Galleria mellonella* as a suitable model of bacterial infection: past, present and future. Front. Cell. Infect. Microbiol..

[22] Coutinho R.L., Visconde M.F., Descio F.J., Nicoletti A.G., Pinto F.C., Silva A.C. (2014). Community-acquired invasive liver abscess syndrome caused by a K1 serotype *Klebsiella pneumoniae* isolate in Brazil: a case report of hypervirulent ST23. Mem.Inst. Oswaldo Cruz..

[23] Godoy R.G., Marcondes M.A., Pessôa R., Nascimento A., Victor J.R., Duarte A. (2020). Bacterial community composition and potential pathogens along the Pinheiros River in the southeast of Brazil. Sci. Rep..

[24] Andrey D.O., Pereira Dantas P., Martins W., Marques De Carvalho F., Almeida L., Sands K. (2020). An emerging clone, *Klebsiella pneumoniae* carbapenemase 2-producing *K. pneumoniae* sequence type 16, associated with high mortality rates in a CC258-endemic setting. Clin. Infect. Dis..

[25] Nascimento T., Cantamessa R., Melo L., Fernandes M.R., Fraga E., Dropa M. (2017). International high-risk clones of *Klebsiella pneumoniae* KPC-2/CC258 and *Escherichia coli* CTX-M-15/CC10 in urban lake waters. Sci. Total Environ..

[26] Bachman M.A., Oyler J.E., Burns S.H., Caza M., Lépine F., Dozois C.M. (2011). *Klebsiella pneumoniae* yersiniabactin promotes respiratory tract infection through evasion of lipocalin 2. Infect. Immun..

[27] Holt K.E., Wertheim H., Zadoks R.N., Baker S., Whitehouse C.A., Dance D. (2015). Genomic analysis of diversity, population structure, virulence, and antimicrobial resistance in *Klebsiella pneumoniae*, an urgent threat to public health. Proc. Natl. Acad. Sci. U. S. A..

[28] De Koster S., Rodriguez Ruiz J.P., Rajakani S.G., Lammens C., Glupczynski Y. (2022). Goossens, *et al*., Diversity in the characteristics of *Klebsiella pneumoniae* ST101 of human, environmental, and animal origin. Front. Microbiol..

[29] Locke J.B., Colvin K.M., Datta A.K., Patel S.K., Naidu N.N., Neely M.N. (2007). *Streptococcus iniae* capsule impairs phagocytic clearance and contributes to virulence in fish. J.Bacteriol..

[30] Patel S., Mathivanan N., Goyal A. (2017). Bacterial adhesins, the pathogenic weapons to trick host defense arsenal. Biomed. Pharmacother..

[31] Wang Z.A., Griffith C.L., Skowyra M.L., Salinas N., Williams M., Maier E.J. (2014). *Cryptococcus neoformans* dual GDP-mannose transporters and their role in biology and virulence. Eukaryot. Cell.

[32] Tamez-Castrellón A.K., van derBeek S.L., López-Ramírez L.A., Martínez-Duncker I., Lozoya-Pérez N.E. (2021). Disruption of protein rhamnosylation affects the *Sporothrix schenckii*-host interaction. Cell. Surf..

[33] McMackin E., Corley J.M., Karash S., Marden J., Wolfgang M.C., Yahr T.L. (2021). Cautionary notes on the use of arabinose- and rhamnose-inducible expression vectors in *Pseudomonas aeruginosa*. J. Bacteriol..

[34] Kawamura T., Ono D., Shirai A., Mimura K., Iida S., Saita K., Oka H., Ohno H. (2022). Acute femoral osteomyelitis due to hypermucoviscous *Klebsiella pneumoniae*. IDCases..

[35] Zhu J., Wang T., Chen L., Du H. (2021). Virulence Factors in Hypervirulent *Klebsiella pneumoniae*. Front. Microbiol..

[36] Liao W., Liu Y., Zhang W. (2020). Virulence evolution, molecular mechanisms of resistance and prevalence of ST11 carbapenem-resistant *Klebsiella pneumoniae* in China: a review over the last 10 years. J. Glob. Antimicrob. Resist..

[37] Oliveira S., Moura R.A., Silva K.C., Pavez M., McCulloch J.A., Dropa M. (2014). Isolation of KPC-2-producing *Klebsiella pneumoniae* strains belonging to the high-risk multiresistant clonal complex 11 (ST437 and ST340) in urban rivers. J. Antimicrob. Chemother..

[38] Teixeira P., Tacão M., Pureza L., Gonçalves J., Silva A., Cruz-Schneider M.P. (2020). Occurrence of carbapenemase-producing Enterobacteriaceae in a Portuguese river: *bla*_NDM_, *bla*_KPC_ and *bla*_GES_ among the detected genes. Environ. Pollut..

[39] Campos-Madueno E.I., Moser A.I., Jost G., Maffioli C., Bodmer T., Perreten V. (2022). Carbapenemase-producing *Klebsiella pneumoniae* strains in Switzerland: human and non-human settings may share high-risk clones. J. Glob. Antimicrob. Resist..

[40] Oliveira P.M., Faria-Junior C., Silva D.M., Matos L.F., Pereira A.L. (2023). Clonal complexes of carbapenem-resistant *Klebsiella pneumoniae* recovered from community sewage. J. Water Health.

[41] Larsson D.G.J., Andremont A., Bengtsson-Palme J., Brandt K.K., de Roda Husman A.M., Fagerstedt P. (2018). Critical knowledge gaps and research needs related to the environmental dimensions of antibiotic resistance. Environ. Int..

[42] Böger B., Surek M., Vilhena R.O., Fachi M.M., Junkert A.M., Santos J.M., Domingos E.L., Cobre A.F., Momade D.R., Pontarolo R. (2021). Occurrence of antibiotics and antibiotic resistant bacteria in subtropical urban rivers in Brazil. J. Hazard. Mater..

[43] Sekizuka T., Yatsu K., Inamine Y., Segawa T., Nishio M., Kishi N., Kuroda M. (2018). Complete genome sequence of a *bla*_KPC-2_-positive *Klebsiella pneumoniae* strain isolated from the effluent of an urban sewage treatment plant in Japan. mSphere..

[44] Cherak Z., Loucif L., Moussi A., Rolain J.M. (2021). Carbapenemase-producing gram-negative bacteria in aquatic environments: a review. J. Glob. Antimicrob. Resist..

[45] Paschoal R.P., Campana E.H., Corrêa L.L., Montezzi L.F., Barrueto L.R.L., da Silva I.R. (2017). Concentration and variety of carbapenemase producers in recreational coastal waters showing distinct levels of pollution. Antimicrob. Agents Chemother..

